# The Potential Protective Effect and Possible Mechanism of Peptides from Oyster (*Crassostrea hongkongensis*) Hydrolysate on Triptolide-Induced Testis Injury in Male Mice

**DOI:** 10.3390/md19100566

**Published:** 2021-10-09

**Authors:** Xueyan Zhang, Zhilan Peng, Huina Zheng, Chaohua Zhang, Haisheng Lin, Xiaoming Qin

**Affiliations:** 1 College of Food Science and Technology, Guangdong Ocean University, Zhanjiang 524088, China; xueyan@stu.gdou.edu.cn (X.Z.); pengzhilan@stu.gdou.edu.cn (Z.P.); zhenghn@gdou.edu.cn (H.Z.); zhangch@gdou.edu.cn (C.Z.); linhs@gdou.edu.cn (H.L.); 2 Guangdong Provincial Key Laboratory of Aquatic Product Processing and Safety, Zhanjiang 524088, China; 3 National Research and Development Branch Center for Shellfish Processing (Zhanjiang), Zhanjiang 524088, China; 4 Guangdong Province Engineering Laboratory for Marine Biological Products, Zhanjiang 524088, China; 5 Guangdong Provincial Engineering Technology Research Center of Marine Food, Zhanjiang 524088, China; 6 Collaborative Innovation Center of Seafood Deep Processing, Dalian Polytechnic University, Dalian 116034, China

**Keywords:** oyster peptides, spermatogenesis, oxidative stress, apoptosis, hormone, testis

## Abstract

Peptides from oyster hydrolysate (OPs) have a variety of biological activities. However, its protective effect and exact mechanism on testicular injury remain poorly understood. This study aimed to evaluate the protective effect of OPs on triptolide (TP)-induced testis damage and spermatogenesis dysfunction and investigate its underlying mechanism. In this work, the TP-induced testis injury model was created while OPs were gavaged in mice for 4 weeks. The results showed that OPs significantly improved the sperm count and motility of mice, and alleviated the seminiferous tubule injury. Further study showed that OPs decreased malonaldehyde (MDA) level and increased antioxidant enzyme (SOD and GPH-Px) activities, attenuating oxidative stress and thereby reducing the number of apoptotic cells in the testis. In addition, OPs improved the activities of enzymes (LDH, ALP and ACP) related to energy metabolism in the testis and restored the serum hormone level of mice to normal. Furthermore, OPs promoted the expression of Nrf2 protein, and then increased the expression of antioxidant enzyme regulatory protein (HO-1 and NQO1) in the testis. OPs inhibited JNK phosphorylation and Bcl-2/Bax-mediated apoptosis. In conclusion, OPs have a protective effect on testicular injury and spermatogenesis disorders caused by TP, suggesting the potential protection of OPs on male reproduction.

## 1. Introduction

Infertility is a universal and serious human health problem, reportedly affecting 8–12% of couples of childbearing age worldwide [1]. Male-related sterility accounts for around 50% of all infertility cases, with approximately 1 in 20 men of reproductive age suffering from infertility [2]. Infertility could be caused by a number of factors, including reproductive system injuries, endocrine disruption, environmental pollution, modern lifestyles, and drug side effects [3,4,5,6,7]. Spermatogenesis occurs in the seminiferous tubules and is strictly dependent on the structure of the testis. Therefore, the integrity of testicular morphological structure and the maintenance of physiological function play important roles in spermatogenesis [8]. Abnormal testicular tissue structure, changes in reproductive hormones, oxidative damage, and cell apoptosis may be the mechanisms of male reproductive dysfunction [9].

Triptolide (TP, C_20_H_24_O_6_), as the main active ingredient of the pharmacological and toxic effects of *Tripterygium wilfordii multiglycoside* (GTW), has long been used in the treatment of inflammatory and immune diseases [10,11,12]. Among the toxic and side effects of TP, its reproductive toxicity leads to the highest incidence of reproductive dysfunction. Excessive triptolide intake interferes with testicular energy metabolism and normal reproductive function, resulting in reduced sperm quality and testicular atrophy, thereby leading to male reproductive dysfunction. Therefore, TP was used as a model drug in the animal models of male sterility to explore the pathogenesis of male sterility and the improvement effect of related drugs on this disease [13].

Oysters are the largest farmed shellfish in the world, rich in protein, glycogen, taurine, and trace elements [14]. In addition to rich nutrition, oyster meat can also affect a variety of physiological functions and has certain health care effects, thus it has been included in the medicine food homology list published by China’s Ministry of Health [15]. As an aquatic product with high protein content, oysters are a good source of polypeptides. Several studies have focused on the health benefits of peptides from oyster hydrolysate (OPs) and have demonstrated their antioxidant [16], immunity-improving [17], antimicrobial [18], antitumor [19], anti-fatigue [20], and liver-protecting [21] properties. In previous studies, Li et al. reported that oyster polysaccharide administration could improve sperm quality and protect reproductive damage in cyclophosphamide-induced male mice [22]. However, the protective effects of OPs on reproductive damage have not been systematically reported. In addition, the study found that OPs may protect the ovary from D-galactose-induced female reproductive dysfunction by reducing oxidative stress, thereby preventing ovarian cell apoptosis [23]. Therefore, OPs have the potential to protect reproductive function against drug damage and deserve further study.

This study aimed to investigate the protective effect of peptides from the oyster hydrolysates on sperm parameters, testicular histopathology, sex hormone levels, activities of testicular marker enzymes, antioxidant level, and cell apoptosis in TP-induced ICR male mice. Furthermore, the activation of OPs on the nuclear factor-erythroid 2-related factor 2 (Nrf2) pathway and inhibition of OPs on the activation of the c-Jun N-terminal kinase (JNK) phosphorylation and Bcl-2/Bax-mediated apoptosis pathway was examined. Eventually, the correlation among the sperm analysis, morphological, biochemical indicators, cell apoptosis, and oxidative stress signaling pathway in the testis was established via the combination of experimental determination and statistical analysis. These findings not only deepen the understanding of OPs against TP-induced testis injury but also provide an experimental basis for the development of OPs as a functional agent.

## 2. Results

### 2.1. Molecular Weight and Main Peptide Sequences of OPs

The function and biological activity of peptides depends on their amino acid composition, sequence, and molecular mass. In this study, 89 peptides with molecular weight ranging from 662.41 to 1590.81 Da were identified by LC-MS/MS, and the peaks of OPs were mainly in the range of 300–800 m/z. As shown in Table 1, the scores for identifying peptide sequences were obtained, and 15 peptide sequences with higher scores were listed.

### 2.2. Amino Acid Composition of OPs

Amino acid composition and content of OPs are presented in Table 2. The contents of total amino acids (TAA) in OPs were 54.75 g/100 g. The essential amino acid (EAA) cannot be synthesized by the body itself and must be obtained through the diet [24]. EAA content of OPs is 22.19 g per 100 g, accounted for 40.53% of TAA, which was highly sufficient to meet the minimum dietary intake of 35% recommended by the World Health Organization [25].

The composition, concentration, and sequence of amino acids have a great influence on the biological activity of proteolytic compounds [20]. The amino acid composition of spermatozoa was significantly changed by amino acid supplementation in the internal environment, which affected sperm motility [26]. Of the 17 amino acids contained in OPs, glutamic acid (8.41 g/100 g), aspartic acid (5.56 g/100 g), arginine (4.23 g/100 g), lysine (4.78 g/100 g), leucine (4.56 g/100 g), and valine (3.34 g/100 g) accounted for a higher proportion. Among them, a diet supplemented with amino acids (mainly lysine, valine, and threonine) improved sperm quality, changed amino acid composition in seminal plasma, and improved sperm motility [27]. Lysine, aspartic acid, and glutamic acid have the ability to chelate metal ions because of the amino or carboxyl groups in their side chains. Hydrophobic amino acids (HAA) accounted for 31.58% of TAA, and its high amounts in OPs offer properties that are able to promote lipid interactions, which enhance entry of the peptides into target organs via hydrophobic associations [28]. BCAAs accounted for 19.95% of TAA, which plays a strong role in maintaining energy supply, and Bahadorani et al. found that appropriate supplementation of BCAAs may have synergistic effects on sperm function and testosterone secretion [24].

### 2.3. Effects of OPs on Sperm Parameters of TP-Induced Mice

**Figure 1:**
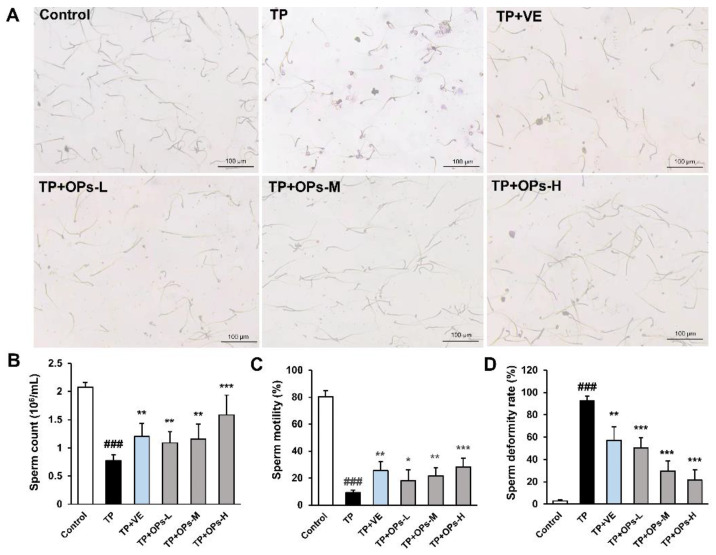
Effects of OPs on sperm quality of ICR mice induced by TP. (**A**) Sperm morphology was observed at 200× magnification; OPs -L, OPs -M, and OPs -H are the groups administrated with 100, 200, and 400 mg/kg OPs separately. (**B**), (**C**), and (**D**) indicate the sperm count, motility, and deformity rate of sperm, respectively. The data were expressed as mean ± SEM, n = 10. Compared with the control group, ### *p* < 0.001; compared with the TP group, * *p* < 0.05, ** *p* < 0.01 and *** *p* < 0.001.

The model of TP-induced mice spermatogenesis dysfunction was established, and mice were treated for 4 weeks. The intervention of TP resulted in severe sperm distortion in mice, and the quantity and quality of sperm were significantly lower than those in the control group. It was even difficult to find an intact normal sperm. As shown in Figure 1A, the sperm morphology of TP-treated mice revealed an increase in acrosome abnormalities: fathead, bent neck, short tail, and coiled-in tail. The treatment of OPs significantly increased the number of normal sperms, elevated the sperm count, improved the sperm motility, and decreased the sperm deformity rate (Figure 1B–D). The effects of different doses of OPs showed a dose dependence, and high dose of OPs treatment significantly ameliorated sperm damage induced by TP (*p* < 0.001).

In this research, VE was chosen as a positive control, based on the fact that its metabolite tocopheryl frequently applied for the promotion of reproductive hormone secretion, increasing sperm numbers and motility, preventing male infertility in the clinic. As shown in Figure 1, VE treatment significantly ameliorated sperm damage induced by TP (*p* < 0.01).

### 2.4. Effects of OPs on Testicular Injury of TP-Induced Mice

**Figure 2:**
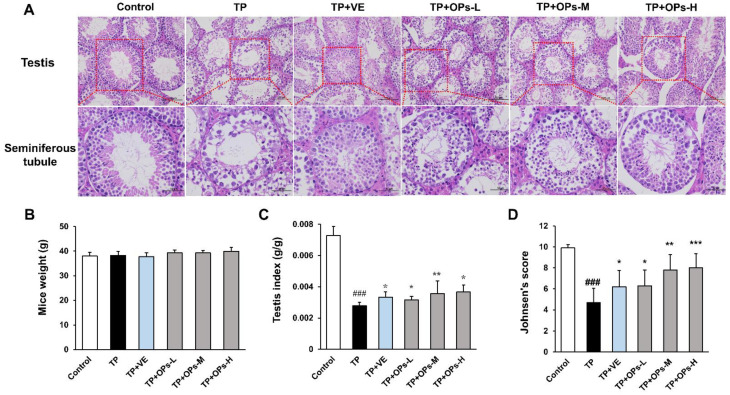
Effects of OPs on testicular injury of ICR mice induced by TP. (**A**) Histopathology with H&E staining (200× and 400×) of the testicular section in mice after treatment for 28 days; (**B**) body weight of mice was measured every 3 days; (**C**) testis index was measured by the ratio of testicular weight to body weight; (**D**) Johnsen’s score in the testicular tissue was determined in each group. The data were expressed as mean ± SEM, n = 10. Compared with the control group, ### *p* < 0.001; compared with the TP group, * *p* < 0.05, ** *p* < 0.01 and *** *p* < 0.001.

The results revealed that there was no significant difference in the bodyweight of mice among the groups (Figure 2B). As shown in Figure 2C, the testis index of the TP group was significantly lower than that in the control group (*p* < 0.001), while treatment of OPs ameliorated the testicular weight loss compared with the TP group. The structure of testicular tissue and the number of testicular cells play an important role in spermatogenesis and sperm quality. The testicular structures and cells play important roles during spermatogenesis, while an ample array of factors can influence its quality and quantity [29]. Histological analysis on the tissue sections of H&E staining showed that OPs treatment protected testis tissue against the damage caused by TP. As compared with controls, TP-induced mice showed severe vacuolation of germ cells, enlarged intercellular spaces, irregular shape, and atrophied seminiferous tubules with only a few Sertoli cells, spermatogonia, and primary spermatocytes (Figure 2A). VE and high-dose OPs (400 mg/kg) treatment restored morphological abnormalities compared with the TP group; vacuolation of germ cells and spermatocyte was decreased. The size of the seminiferous tubule, the layer of the spermatocytes, and the number of Sertoli cells were preserved by the treatment of VE and high-dose OPs (Figure 2A). The overall structures of the seminiferous tubule in testis were evaluated by Johnsen’s scoring method. Compared with the TP group, the middle and high dose of OPs elevated Johnsen’s score significantly (*p* < 0.01) (Figure 2D).

### 2.5. Effects of OPs on TP-Induced Reproductive Hormone Level

**Figure 3:**
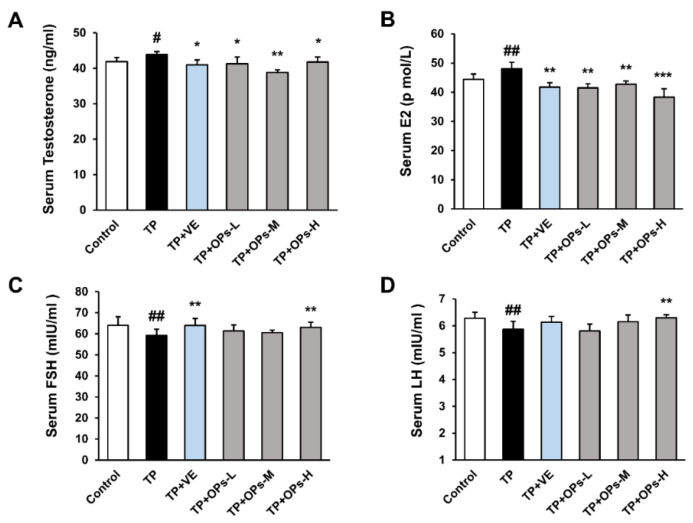
Effects of OPs on serum reproductive hormone level of ICR mice induced by TP. (**A**) Serum testosterone level, (**B**) serum estradiol (E2) level, (**C**) serum follicle stimulating hormone (FSH) level, (**D**) serum luteinizing hormone (LH) level. The data were expressed as mean ± SEM, n = 10. Compared with the control group, # *p* < 0.05 and ## *p* < 0.01; compared with the TP group, * *p* < 0.05, ** *p* < 0.01 and *** *p* < 0.001.

Male reproductive hormones regulate the process of spermatogenesis. As shown in Figure 3, the concentration of serum testosterone and estradiol was significantly increased in the TP group, and the levels of follicle stimulating hormone (FSH) and luteinizing hormone (LH) were significantly decreased compared with the control group (*p* < 0.01). Obviously, it was shown that TP disturbed the serum hormone level of mice compared with the controls. As compared with the TP group, the treatment of OPs restored the serum testosterone and estradiol level of mice, which were close to the control group. Additionally, a significant increase in FSH and LH levels was seen in the TP+OPs-H group compared with the TP group (*p* < 0.01). VE as a positive control significantly restored the TP-induced serum reproductive hormone (testosterone, estradiol, and FSH) disorder in mice.

### 2.6. OPs Increased Testicular Marker Enzyme Activity and Reduced Oxidative Stress Induced by TP

**Figure 4:**
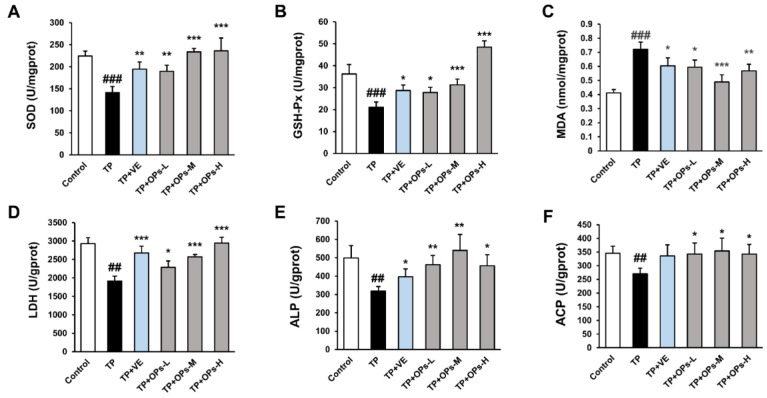
Effects of OPs on testicular marker enzymes and bio markers of oxidative stress in testes tissues of ICR mice induced by TP. (**A**) superoxide dismutase (SOD) level, (**B**) glutathione peroxidase (GSH-Px) level, (**C**) malondialdehyde (MDA) level, (**D**) lactate dehydrogenase (LDH) level, (**E**) alkaline phosphatase (ALP) level, (**F**) acid phosphatase (ACP) level. The data were expressed as mean ± SEM, n = 10. Compared with the control group, ## *p* < 0.01 and ### *p* < 0.001; compared with the TP group, * *p* < 0.05, ** *p* < 0.01 and *** *p* < 0.001.

Oxidative stress is a cause of testis injury which was also found in the TP-induced mice model (Figure 4A–C). TP raised the lipid peroxidation product malondialdehyde (MDA) and disrupted the antioxidative system of the testis, including the enzyme superoxide dismutase (SOD) and glutathione peroxidase (GSH-Px). OPs treatment reduced the level of MDA and increased the activity of SOD and GSH-Px, and especially the middle and high two-dose groups of OPs showed extremely significant changes (*p* < 0.001). Meanwhile, VE treatment as a positive control also significantly reduced oxidative stress in testicular tissue induced by TP.

Activities of testicular marker enzymes such as lactate dehydrogenase (LDH), acid phosphatase (ACP) level, and alkaline phosphatase (ALP) are considered as functional indicators of spermatogenesis and testicular development. The alterations of testicular marker enzyme activity could affect the energy metabolism pathway, thus interfering with the energy supply of spermatogenesis. As were shown in Figure 4D–F, TP significantly suppressed the level of testicular marker enzymes in testis tissue, including LDH, ALP, and ACP (*p* < 0.01). As compared with the TP group, middle and high doses of OPs significantly increased the activity of LDH (*p* < 0.001), and different dose of OPs ameliorated the activity of ALP and ACP. Meanwhile, VE treatment significantly increased the activity of testicular marker enzymes (LDH and ALP).

### 2.7. Effects of OPs on the Testicular Apoptotic Induced by TP

**Figure 5:**
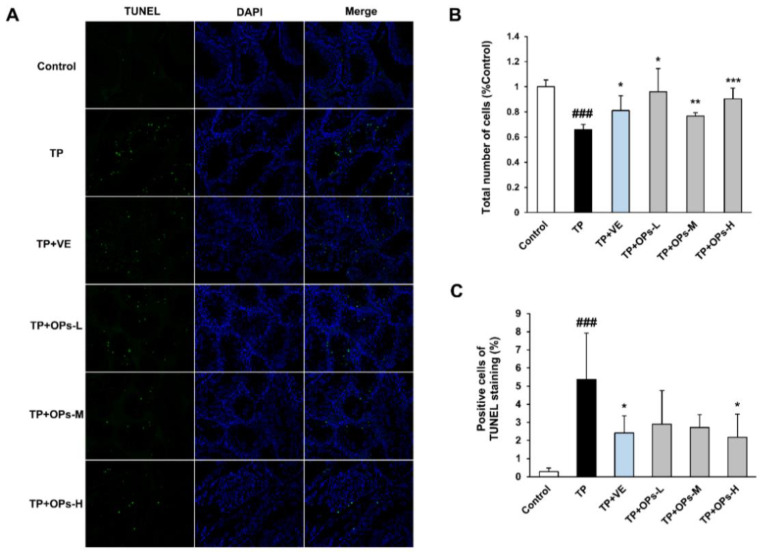
Effects of OPs on apoptotic index in testes tissues of ICR mice induced by TP. (**A**) Apoptotic cells in testicular tissue sections were detected by TUNEL assay (apoptotic cells: green fluorescence, cell nucleus: blue fluorescence). (**B**) The total number of cells in each group compared by Control group were shown. (**C**) The ratios of apoptotic cell were shown. The data were expressed as mean ± SEM, n = 3. Compared with the control group, ### *p* < 0.001; compared with the TP group, * *p* < 0.05, ** *p* < 0.01 and *** *p* < 0.001.

The results of terminal deoxynucleotidyl transferase dUTP nick-end labeling (TUNEL) staining of cell apoptosis were shown in Figure 5. There were few apoptotic cells in the testicular tissue of the control group, which is common during spermatogenesis. However, in the TP group, the proportion of the apoptotic cells was significantly increased despite the loss of total cells in seminiferous tubules (Figure 5B,C). VE and OPs treatment significantly ameliorated the decrease in the total number of cells in seminiferous tubules induced by TP (Figure 5B). Meanwhile, VE and high dose of OPs treatment reduced the number and proportion of the apoptotic cells (Figure 5C).

**Figure 6:**
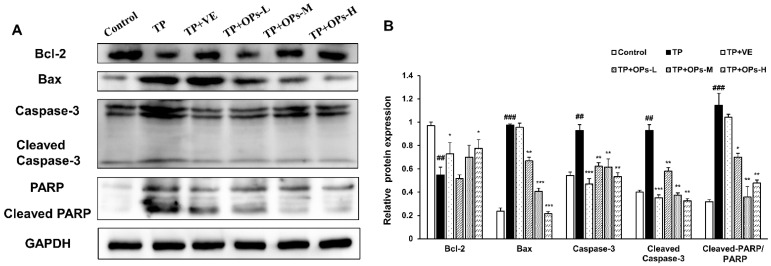
Effects of OPs on protein expression levels of markers of apoptosis in TP-induced testis tissues of male ICR mice. (**A**) Electrophoresis images of Bcl-2, Bax, Caspase-3, Cleaved Caspase-3, PARP, and GAPDH protein; (**B**) the quantitative densitometric analysis of Bcl-2, Bax, Caspase-3, Cleaved Caspase-3, and PARP proteins. The data were expressed as mean ± SEM, n = 3. Compared with the control group, ## *p* < 0.01 and ### *p* < 0.001; compared with the TP group, * *p* < 0.05, ** *p* < 0.01 and *** *p* < 0.001.

To further elucidate the testicular apoptotic events, the protein expression of Bcl2, Bax, caspase-3, and PARP reliable apoptotic markers were analyzed. As shown in Figure 6, TP stimulated the upregulation of Bax, caspase-3, cleaved caspase-3, and cleaved PARP, and inhibited the expression of Bcl-2. The upregulated Bax, caspase-3, cleaved caspase-3, and cleaved PARP were significantly inhibited by treatment of OPs at three doses. VE treatment downregulated the activation of cleaved caspase-3. The decrease in Bcl-2 expression was significantly upregulated by VE and high dose of OPs treatment. These findings suggested that OPs ameliorated TP-induced apoptosis in testicular tissue.

### 2.8. Effects of OPs on Related Proteins Expression in the Nrf2 and JNK Pathways

**Figure 7:**
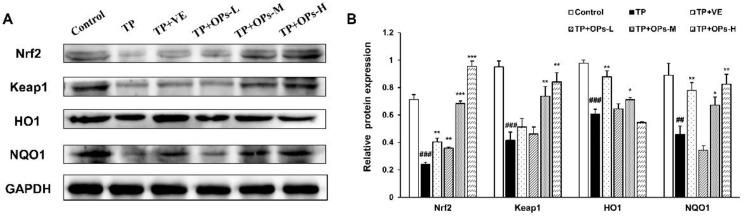
Effects of OPs on the Nrf2/Keap1 signaling pathway in TP-induced testis tissues of male ICR mice. (**A**) Electrophoresis images of Nrf2, Keap1, HO-1, NQO1, and GAPDH protein; (**B**) The quantitative densitometric analysis of Nrf2, Keap1, HO-1, and NQO1 proteins. The data were expressed as mean ± SEM, n = 3. Compared with the control group, ## *p* < 0.01 and ### *p* < 0.001; compared with the TP group, * *p* < 0.05, ** *p* < 0.01 and *** *p* < 0.001.

**Figure 8:**
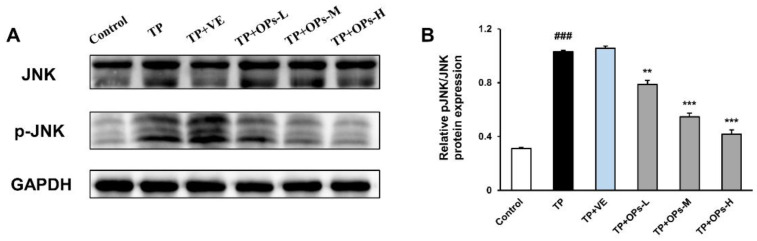
Effects of OPs on the JNK signaling pathway in TP-induced testis tissues of male ICR mice. (**A**) Electrophoresis images of JNK, p-JNK and GAPDH protein; (**B**) the quantitative densitometric analysis of p-JNK/JNK bands. The data were expressed as mean ± SEM, n = 3. Compared with the control group, ### *p* < 0.001; compared with the TP group, ** *p* < 0.01 and *** *p* < 0.001.

Detection of the levels of related protein factors in the testicular tissue via Western blot was illustrated in Figure 7 and Figure 8. As displayed in Figure 7, administration of TP led to the downregulation of Nrf2, Keap1, HO-1, and NQO1 expression in mice testis tissues, concomitant with upregulated expressions of p-JNK and p-JNK/JNK, and total JNK protein expression was not affected by TP treatment, as shown in Figure 8. ImageJ software was used to obtain optical density values of the protein bands. The decrease in Nrf2, Keap1, HO1, and NQO1 protein expression were upregulated by treatment of OPs at middle and high doses. VE as a positive control upregulated the Nrf2, HO1, and NQO1 protein expression. The treatment with OPs downregulated the activation of p-JNK to a nearly normal level.

## 3. Discussion

In this study, male infertility disease model mice were successfully established by TP induction. In the model, the deteriorated sperm quality, altered testicle histomorphology, disordered hormone levels, the decreased activity of testicular marker enzyme, and the triggered testis oxidative stress and germ cells apoptosis were observed. The observed altered sperm quality and testicle histomorphology in this study is consistent with the previous reports [8,13,30]. Gastric administration of appropriate dose of OPs could reverse these abnormalities. The results showed that OPs treatment in TP-induced mice has beneficial effects on the testis index, sperm parameters, histological structure of testis, hormone level, testicular marker enzyme activity, and oxidative stress, as well as testicular apoptosis. The mechanism of the protective effects of OPs may be through inhibiting the oxidative stress by Nrf2 and JNK pathways, increasing the expression of Bcl2, and reducing the level of cell apoptosis by suppressing the expression of the apoptotic markers Bax, caspase-3, and PARP.

Oxidative stress due to toxic substances is considered to be closely related to male infertility [31]. Production of reactive oxygen species (ROS) and consequent oxidative damage have been established as mechanisms for TP toxicity [13]. It is well known that oxidative stress leads to sperm dysfunction by inducing peroxidation damage to the plasma membrane. In this study, TP-induced increased MDA levels and decreased GSH-Px and SOD levels in mice, suggesting oxidative stress, which was consistent with the reports of some researchers [8,13,32]. As spermatozoa are high in polyunsaturated fatty acids (PUFAs), it is more sensitive to oxidative damage than other cells [33]. Furthermore, the generation of ROS decreased the number of spermatogonia cells in the testis, and it is thought to be detrimental for spermatogenesis [34]. In this study, the sperm count and motility of mice were significantly decreased, and the sperm deformity rate was increased, induced by TP. The significant reduction in spermatogonia was also observed on histopathological assay. The severe impairment of sperm characteristics induced by TP is closely related to the obvious oxidant/antioxidant imbalance in testis. The treatment of OPs effectively reduced the deterioration of sperm quality, which may be related to the amelioration of oxidative stress in testicular microenvironment as manifested by decreasing in the level of MDA and increasing in the activity of SOD and GSH-Px. It suggested that OPs exert significant antioxidant effects in testicular tissue, including the increase in activities of antioxidant enzymes and decrease in lipid peroxidation. It has been reported that peptides (2-20 amino acids) can completely cross the intestinal barrier and perform biological functions in tissues, while peptides the size of 5–16 amino acids show potent antioxidant activity [28,35], which is manifested in its ability to carry out DPPH (2,2-diphenyl-1-picrylhydrazyl) free radical scavenging [16,20]. Combined with the results of animal experiments, the main peptides of OPs with 6–14 amino acid residues in length may also show antioxidant activity, suggesting that its alleviation of ROS-induced testicular injury may be related to its antioxidant function. In addition, compared with other hydrophilic amino acids, hydrophobic amino acids have higher antioxidant activity in peptides, and its high amounts in OPs may play an important role in reducing the oxidative damage of the testis caused by ROS [36].

Decreased activity of antioxidant enzymes could induce the accumulation of ROS and lead to oxidative stress. The expression of antioxidant enzymes including HO-1, NQO1, and SOD were mainly mediated by the Nrf2 pathway. Under normal conditions, Nrf2 is mainly regulated by the repressor protein Keap1 in the cell cytoplasm. The disturbance of interaction between Nrf2 and Keap1 or degradation of Keap1 affect the further activation of downstream antioxidant enzyme expression by Nrf2 [37]. Recent studies have shown that the regulation of endogenous antioxidant system through the Nrf2 pathway significantly reduces oxidative stress-induced apoptosis of Sertoli cells and testicular injury [32]. In this study, TP-induced decrease the expression of NQO1 and HO-1 protein by decreasing Nrf2 and Keap1 expression was consistent with the results of the previous study [8]. Pretreatment with OPs may inhibit the decrease in antioxidant enzyme expression by activating Nrf2 expression. This finding suggests that the protective effect of OPs on testicular injury might be related to the upregulation of Nrf2 expression.

Previous studies showed that TP could promote the production of ROS in Sertoli cells, thus further activating the JNK pathway, which triggered the mitochondrial-mediated apoptosis pathway [13]. In vivo results showed that TP reduced testicular weight, destroyed the microstructure of the testis, disrupted the enzyme activity, increased MDA levels, activated JNK phosphorylation, and promoted testicular tissue apoptosis. In this study, treatment of OPs significantly reduced the activation of JNK phosphorylation, which indicated that OPs may protect testis against oxidative stress by mediating the JNK pathway.

Mitochondria are responsible for cells energy metabolism and are the main targets of ROS production and regulation of apoptosis. In our study, it was found that OPs may reduce the excessive apoptosis of testicular cells mediated by the mitochondrial apoptosis pathway, as shown in the results of TUNEL assay and the Western blotting of apoptosis-related proteins (Bax, Bcl-2, caspase-3, and PARP). Both the anti-apoptotic protein Bcl-2 and the pro-apoptotic protein Bax regulate apoptosis by controlling the permeability of the mitochondrial membrane. OPs treatment significantly upregulated the expression of Bcl-2, downregulated the expression of Bax, thereby regulating cell permeability, further inhibiting the activation of Caspase-3 and reducing the Caspase cascade reaction. Several studies have shown that increased ROS accumulation may lead to increased permeability of the mitochondrial outer membrane and then activate mitochondria-dependent apoptosis signaling pathway to induce cell apoptosis. At present, few studies have shown that OPs play a direct role in the inhibition of cell apoptosis, while in the activation of the apoptosis process, it is believed that oxidative stress-induced damage can trigger apoptotic signaling procedures, leading to cell death. Therefore, it indicated that OPs may mediate mitochondria-dependent apoptosis signaling pathways to reduce cell apoptosis and tissue damage by reducing oxidative damage of mitochondrial lipids caused by ROS. In our study, it was also showed that OPs reversed severe testicular tissue damage by ameliorating testicular apoptosis by assessing the testis histopathology.

Sertoli cells, as nursing cells, regulate the processes of spermatogenesis by providing nutritional support and a suitable situation for the survival and development of germ cells [38]. The altered testicular enzyme activities induced by TP may have resulted from the impairment of Sertoli cell function and disrupted metabolism in mice [39]. LDH enzyme is involved in the process of glycolysis and gluconeogenesis and controls the synthesis of the main energy source of germ cells. The ACP enzyme is mainly distributed in the cytoplasm of Sertoli cells, which is involved in protein synthesis and related to the phagocytosis of Sertoli cells. ALP is involved in the synthesis of nucleic acids, nucleoproteins, and phospholipids, such as in the cleavage of phosphate esters and in mobilizing carbohydrates and lipid metabolites to be used by spermatozoa [40]. In this study, TP inhibited the activities of LDH, ACP, and ALP, which not only interfered with the energy supply process of aerobic and anaerobic glycolysis but also disturbed the energy utilization of testis. OPs treatment improved the energy metabolism of testicular tissue and the energy supply of spermatogenesis by increasing the enzyme activity of LDH, ACP, and ALP. In addition, metabolic disorders may be due to the disintegration of mitochondrial membrane ultrastructure caused by mitochondrial lipid peroxidation. It has been found that lipid peroxidation induced by oxidative stress produces a strong cytotoxic effect in the testis, leading to the damage of nucleic acid, protein, carbohydrates, and lipids in cells. Spermatogenesis is reduced due to the resulting disturbances in energy metabolism, oxidative phosphorylation, tricarboxylic acid cycle, and glycolysis. Previous studies have shown that OPs could protect TM4 Sertoli cells from toxic damage induced by TP, including improving the cell viability of TM4 cells, reducing the production of intracellular ROS and lipid peroxidation, and enhancing the antioxidant activity of TM4 cells [41]. Combined with the results of this study, it was found that OPs ameliorated TP-induced metabolic disorders by inhibiting mitochondrial lipid peroxidation.

Spermatogenesis is a multistep process that could be disturbed by multifaceted factors. Expect for oxidative stress, hormone imbalance can affect the process of spermatogenesis [42]. Destructive endogenous hormone signaling might mediate the process of spermatogenesis and lead to low sperm count [43]. LH and FSH are considered to be key factors in the regulation of testis function. LH is related to produce testosterone by stimulating the Leydig cells and both FSH and testosterone in turn regulate Leydig cells activity and stimulate germ cell proliferation and differentiation by stimulating the Sertoli cells. Estradiol is converted from circulating testosterone by enzyme aromatase; therefore, its concentration is affected by the level of testosterone. However, dysregulation of circulating estradiol may lead to inhibition of LH production not beneficial for spermatogenesis [44]. In this study, TP increased the levels of serum testosterone and estradiol, and decreased serum LH and FSH levels in mice. The treatment of OPs restored the serum hormone level of mice to the normal, and reduced the disturbance of hormones on spermatogenesis in mice. Additionally, previous studies had found that oyster peptides improved the level of serum androgen induced by cyclophosphamide [45].

The identification of the active components of oyster peptides is of great interest in exploring new strategies for the treatment or prevention of testicular and sperm damage. Previous studies showed that some antioxidant peptides were identified from oysters, such as Pro-Val-Met-Gly-Asp, Glu-His-Gly-Val, and Leu-Lys-Gln-Glu-Leu-Glu-Asp-Leu-Leu-Glu-Lys-Gln-Glu [16,46]. These antioxidant peptides may play an important role in preventing oxidative damage in the testis, resulting in reduced sperm quality. However, other active ingredients still need to be identified by Sephadex gel chromatography, HPLC, mass spectrometry, and other purification methods.

**Figure 9:**
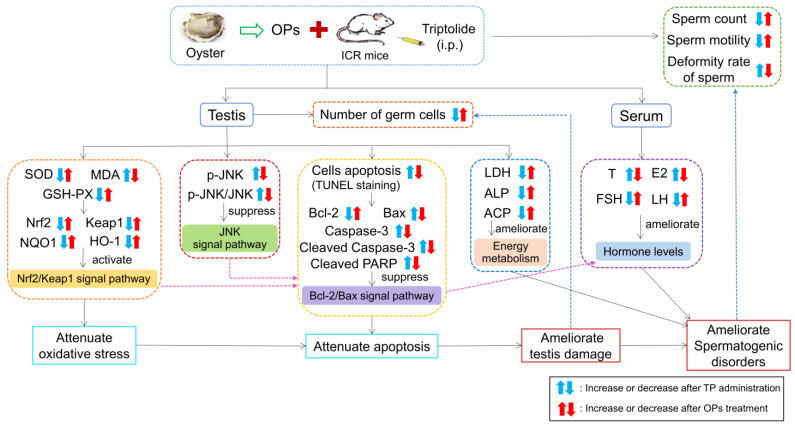
Possible mechanism underlying the protective effects of OPs intervention on TP-induced testicular damage in mice. By detecting MDA, antioxidant enzymes and related proteins in the testis, as well as sexual hormone levels in serum, the anti-effect of OPs was shown to be related to Nrf2/Keap1, JNK, and Bcl-2/Bax pathways.

In conclusion, the underlying mechanism of OPs in the potential protective effect on testis and sperm is attributed to the synergistic modulations, and these results deepen the understanding of the potential improvement of male reproductive function by OPs, thus demonstrating the potential application of OPs in functional foods (Figure 9).

## 4. Materials and Methods

### 4.1. Materials and Reagents

Fresh oysters (*Crassostrea hongkongensis*) were purchased from the local market in Zhanjiang, China (oysters were shelled at the market and immediately stored at −30 °C until use). Compound protease (4.5 × 10^5^ U g^−1^) was obtained from Pangbo Biotech (Nanjing, China). Triptolide (purity > 98%) were purchased from Meilun-Biotech Co., Ltd. (Dalian, China). Vitamin E (VE) (H44021026) was purchased from Baiyunshan Pharmaceutical Factory (Guangzhou, China). All chemicals and reagents used were of analytical grade and commercially available.

### 4.2. Preparation of OPs

OPs were prepared by enzymatic hydrolysis from the oyster meat according to the methods of Li et al., Peng et al., and Zhang et al. [17,41,47]. Briefly, three kilograms of oyster meat were ground into mince and then mixed with distilled water (1:3 *w*/*v*). The mixture was homogenized at 8000 rpm for 5 min by using a homogenizer. Homogenates were hydrolyzed at pH 7.0 with compound protease (enzyme concentration 1000 U g^−1^ of raw material). The hydrolysis reaction lasted for 5 h in a 53 °C water bath. Subsequently, the protease was inactivated at 100 °C for 10 min and the enzymatic hydrolysate was centrifuged at 15,000g at 4 °C for 20 min to obtain the supernatant. The supernatant was fractionated by an ultrafiltration device (XX42PMINI, Millipore, USA) and 10 kDa, 5 kDa, and 3 kDa ultrafiltration membranes (Mili Pellicon, Millipore, USA) to obtain the components used in this study (< 3 kDa hydrolysate fraction). The samples were freeze-dried into powder for subsequent experiments (FD-551, EYELA, Tokyo, Japan).

### 4.3. OPs Characterization by LC-ESI/MS/MS

The molecular weight and amino acid sequences of the OPs were identified by liquid chromatography–electrospray ionization tandem mass spectrometry (LC-ESI/MS/MS). The sample (1 μL) was injected with an autosampler and subsequently separated by a C18 column and eluted with conditions of mobile phase A (H_2_O, 0.1% formic acid) and mobile phase B (95% acetonitrile, 0.1% formic acid). Peptides were first eluted with a linear gradient from 2% to 35% of mobile phase B for 40 min, and then from 35% to 80% of mobile phase B for 10 min, running temperature of 25 °C at a flow rate of 0.3 μL/min. After chromatography, ESI-MS/MS was carried out using a Q-EXACTIVE mass spectrometer (Thermo Fisher Scientific, San Jose, CA) equipped with the electrospray ionization (ESI) source. The spectrometer worked in the positive ion mode, and the parameters of the ESI ionization interface were set as follows: capillary voltage 1.6 kV, Resolution 70,000, AGC target:1e5, NCE/stepped NCE: 27. Samples were analyzed with a full-scan MS mode in the range of 350–2000 m/z to obtain the total ion chromatogram. Then, the Mascot search engine was used to analyze the chromatographic peaks corresponding to the mass spectrometry of the custom oyster protein database. Only peptides identified with *p*< 0.05 significance were recorded.

### 4.4. Determination of Amino Acid Composition

The amino acid composition and content of OPs were determined by an amino acid autoanalyzer (L-8900, Hitachi, Tokyo, Japan). The sample and 6 mol/L HCl were added into a tube containing phenol for hydrolysis. After the tube was vacuumed, the mixture was washed with nitrogen and hydrolyzed at 110 °C for 22 h. After the filtrate had cooled, it was heated to 40–50 °C with a test tube concentrator and dried under reduced pressure until evaporated. Sodium citrate buffer solution was added to the dried test tube and dissolved. The solution was transferred to the injection flask of the instrument for determination by the amino acid analyzer [47]. According to the peak area in comparison with the standard, amino acid contents were calculated.

### 4.5. Animals and Treatment

The Laboratory Animal Committee of Guangdong Ocean University, China (no. GDOU-LAE-2020-014) approved all the animal protocols and experimental procedures for this study. Sixty adult ICR male mice (specific pathogen-free, approval no.11032201101487454) were purchased from the Huafukang Biotech Co., Ltd. (Beijing, China) and acclimatized for 7 days before the start of the experiments. All mice were housed at 4–5 mice per cage (23 cm × 32 cm × 15 cm) and randomly fed a normal diet in an environmentally controlled room (temperature was 25 ± 2 °C and relative humidity was 50–65%, with a 12 h light/dark cycle). Animals were randomly divided into six groups (n = 10). Control group: mice were given 0.9% NaCl orally and intraperitoneally, once a day; TP (model group): mice were pretreated with 0.9% NaCl and intraperitoneally injected with TP at a dose of 120 μg/kg; TP + VE (positive control group): mice were orally administrated with VE at a dose of 7.5 mg/kg, and intraperitoneally injected with TP (120 μg/kg) after 1 h every day; TP + OPs: Mice were orally administrated with OPs at the dose of 100 mg/kg (TP + OPs-L), 200 mg/kg (TP + OPs-M), 400 mg/kg (TP + OPs-H) and intraperitoneally injected with TP (120 μg/kg) after 1 h every day. Administration methods and doses of TP-induced testicular injury were conducted according to Wang et al., Ma et al., and Zhang et al. [8,13,32]. VE was determined as the positive control drug according to the study results of Li et al. and Hamza et al. [22,48]. After 28 days of continuous treatment, mice were sacrificed by enucleation of the eye and bled for the collection of blood, and all testes were removed and weighted to measure the testis index (testis weight/body weight). The right epididymis of mice was collected for sperm analysis, and the right testis of mice was stored at -80 °C for protein expression and other parameter detection. The left testis was fixed in 4% paraformaldehyde for further histological analysis.

### 4.6. Sperm Analysis

The entire right epididymis was placed in 1 mL of prewarmed saline and minced into small sections, incubating for 10 min (37 °C, 5% CO_2_) to allow the spermatozoa to swim out from the epididymal tubules. The sperm suspension was dropped into a Neobar’s hemocytometer, and the sperm count was estimated under a cover glass. The sperm motility was observed under a 400× light microscope (Olympus Corporation, Tokyo, Japan) and calculated and expressed as a percentage of motile sperm according to the World Health Organization manual criteria. The sperms were stained using the Quick sperm stain kit (Nanjing Jiancheng Bioengineering Institute, Nanjing, China) according to the manufacturer’s instructions. Additionally, images of sperm after staining were observed under a light microscope and acquired by photographing using a Leica DMI4000B microscope with Leica DFd4500 imaging system (Leica Corporation, German). The rate of abnormal morphology of sperm was evaluated and calculated by observing sperm staining images. Sperm parameters of mice (count, motility, and morphology) were analyzed in each group according to the methods adopted by Qiu et al. and Oghbaei et al. [49,50].

### 4.7. Histopathological and Ultrastructural Assessment

The left testes of mice dehydrated in gradient concentrations of alcohol, and cleared with xylene, then embedded in paraffin at 65 °C. After cooling at −20 °C, the sections were cut at 4 μm and stained with hematoxylin and eosin (H&E). The sections of testis tissue were observed under a light microscope and photographed at magnifications of 200× and 400× using a Nikon Eclipse E100 microscope with Nikon DS-U3 imaging system (Nikon Corporation, Japan). The testicular structure of each animal was assessed, and Johnsen’s score was assessed based on spermatogenic function and germ cell count from 1 (no spermatogenic epithelium) to 10 (normal spermatogenesis). All analyses were performed by an evaluator who was unaware of the treatment group.

### 4.8. Measurements of Enzyme and Hormone

The homogenates of right testis tissue were prepared to determine the levels of testicular superoxide dismutase (SOD), glutathione peroxidase (GSH-Px), malondialdehyde (MDA), and testicular marker enzymes containing lactate dehydrogenase (LDH), alkaline phosphatase (ALP), and acid phosphatase (ACP) according to the specific steps in the kit instructions (Nanjing Jiancheng Bioengineering Institute, Nanjing, China). The protein concentrations of testicular tissue homogenates were determined by the bicinchoninic acid (BCA) protein assay kit (Beyotime Biotechnology, Shanghai, China). Sex hormones of mice including testosterone (T), estradiol (E2), follicle-stimulating hormone (FSH), and luteinizing hormone (LH) in serum were measured by commercial enzyme-linked immunosorbent assay (ELISA) kits (Mmbio, Jiangsu, China) following the instructions.

### 4.9. TUNEL Apoptosis Assay

The embedded wax block sections were dewaxed in water and treated with proteinase K and rupturing cell membrane working solution at 37 °C for 22 min, then washed with PBS at pH 7.4 three times. The mixture of TDT and DUTP (1:5) was incubated at 37 °C for 2 h, followed by 4’,6-diamidino-2-phenylindole (DAPI) staining for 10 min after washing. The results were observed under a Nikon Eclipse C1 fluorescence microscope with Nikon DS-U3 imaging system (Nikon Corporation, Japan) and the images were collected. At a magnification of 200×, the positive cells of TUNEL staining were counted in 4 random fields on each section.

### 4.10. Western Blotting

Testicular tissues were homogenized over ice using a homogenizer with 1% protease inhibitor cocktail (Service bio). Then, after repeated mixing for 30 min in ice, the supernatant was collected after centrifugation at 12,000g and 4 °C for 10 min. Total protein concentrations were determined by a BCA protein assay kit (Service bio) and adjusted to the same level. A total of 30 µg of protein extracts from testicular issues were separated by 5% SDS-polyacrylamide gel electrophoresis (SDS-PAGE) and transferred to polyvinylidene fluoride (PVDF) membranes (Millipore, USA), and followed by blocking with 5% skimmed milk at room temperature for 1 h. The primary antibodies were added into the membrane and then incubated overnight at 4 °C. Primary antibodies to the following proteins were used: Keap1 (sc-514914; 1:500), Heme Oxygenase 1 (sc-390991; 1:1000), NQO1 (sc-376023; 1:1000), Bax (sc-20067; 1:1000), JNK (sc-7345; 1:500), p-JNK (sc-6254; 1:500), cleaved PARP (sc-56196; 1:1000) (Santa Cruz, CA) and Nrf2(16396-1-AP; 1:1000), Bcl2 (26593-1-AP; 1:1000), Caspase-3 (19577-1-AP; 1:1000) (Proteintech, USA). After washing, the membranes were incubated with secondary antibodies at 1:2000 at room temperature for 2 h, and the marked proteins were illuminated using ECL Reagent Kit (Service bio). The bands were visualized by the scanner EPSON V300 (Seiko Epson Corporation, Japan) as well as its intensity was analyzed by ImageJ software and normalized to GAPDH levels.

### 4.11. Statistical Analysis

Results were expressed as the mean ± standard deviation (SD) and analyzed by SPSS version 17. All the experimental data were analyzed using One-way ANOVA. Differences at *p* < 0.05 was considered statistically significant.

## Figures and Tables

**Table 1:** Main peptide sequences of OPs.

No.	Peptide Sequence	Theoretical Mass (Mr)	Observed Mass (m/z)	Scores
1	LAGPQSIIGRTM	1242.68	622.34	56.04
2	IIDAPGHRDF	1139.57	380.87	62.28
3	YDNEFGYSFR	1296.55	649.28	56.59
4	RVPVPDVSVVDL	1293.73	647.87	48.44
5	AFRVPVPDVSVVDL	1511.84	756.93	37.93
6	GIVLDSGDGVSH	1154.56	578.28	57.46
7	LDLAGRDLTD	1087.56	544.78	36.34
8	PDGQVITI	841.46	421.73	35.7
9	KSYELPDGQVIT	1348.69	675.35	33.95
10	KSYELPDGQVITIG	1518.79	760.4	33.88
11	IAQDFKTDLR	1205.65	402.89	37.72
12	GLALLVP	681.45	341.73	32.1
13	LLQALD	671.39	336.7	35.92
14	GIVLDSGDGVTH	1168.58	585.3	42.1
15	LDLAGRDLTD	1087.55	544.78	36.34

**Table 2:** The amino acids composition of OPs (g/100 g).

Amino Acid	Contents of OPs
Thr	2.81
Val	3.34
Met	1.41
Ile	3.02
Leu	4.56
Phe	2.27
Lys	4.78
His	1.03
Arg	4.23
Asp	5.56
Ser	2.88
Glu	8.41
Pro	2.30
Gly	3.25
Ala	2.69
Tyr	2.21
Cys	ND ^e^
TAA ^a^	54.75
EAA ^b^	22.19
HAA ^c^	17.29
BCAA ^d^	10.92

^a^ TAA: Total amino acids. ^b^ Essential amino acid (EAA): Thr, Val, Met, Ile, Leu, Phe, Lys. ^c^ Hydrophobic amino acids (HAA): Ala, Leu, Ile, Met, Phe, Pro, Tyr, and Val. ^d^ Branched-chain amino acids (BCAA) = Leu, Ile, and Val. ^e^ ND: not detected.

## Data Availability

Data are available upon request.

## References

[1] Inhorn M.C., Patrizio P. (2015). Infertility around the globe: New thinking on gender, reproductive technologies and global movements in the 21st century. Hum. Reprod. Update.

[2] Jarow J.P., Sharlip I.D., Belker A.M., Lipshultz L.I., Sigman M., Thomas A.J., Schlegel P.N., Howards S.S., Nehra A., Damewood M.D. (2002). Best practice policies for male infertility. J. Urol..

[3] Karna K.K., Choi B.R., Kim M.-J., Kim H.K., Park J.K. (2019). The Effect of Schisandra chinensis Baillon on Cross-Talk between Oxidative Stress, Endoplasmic Reticulum Stress, and Mitochondrial Signaling Pathway in Testes of Varicocele-Induced SD Rat. Int. J. Mol. Sci..

[4] Benoff S., Jacob A., Hurley I.R. (2000). Male infertility and environmental exposure to lead and cadmium. Hum. Reprod. Update.

[5] Clavijo R.I., Hsiao W. (2018). Update on male reproductive endocrinology. Transl. Androl. Urol..

[6] Ilacqua A., Izzo G., Emerenziani G.P., Baldari C., Aversa A. (2018). Lifestyle and fertility: The influence of stress and quality of life on male fertility. Reprod. Biol. Endocrinol..

[7] Semet M., Paci M., Saïas-Magnan J., Metzler-Guillemain C., Boissier R., Lejeune H., Perrin J. (2017). The impact of drugs on male fertility: A review. Andrology.

[8] Ma B., Zhang J., Zhu Z., Bao X., Zhang M., Ren C., Zhang Q. (2019). Aucubin, a natural iridoid glucoside, attenuates oxidative stress-induced testis injury by inhibiting JNK and CHOP activation via Nrf2 up-regulation. Phytomedicine.

[9] Liu F.J., Dong W.Y., Zhao H., Shi X.H., Zhang Y.L. (2019). Effect of molybdenum on reproductive function of male mice treated with busulfan. Theriogenology.

[10] Kupchan S.M., Court W.A., Dailey R.G., Gilmore C.J., Bryan R.F. (1972). Triptolide and tripdiolide, novel antileukemic diterpenoid triepoxides from Tripterygium wilfordii. J. Am. Chem. Soc..

[11] Wang B., Ma L., Tao X., Lipsky P.E. (2004). Triptolide, an active component of the Chinese herbal remedy Tripterygium wilfordii Hook F, Inhibits production of nitric oxide by decreasing inducible nitric oxide synthase gene transcription. Arthritis Rheum..

[12] Lu Y., Bao X., Sun T., Xu J., Zheng W., Shen P. (2012). Triptolide attenuate the oxidative stress induced by LPS/D-GalN in mice. J. Cell Biochem..

[13] Wang Y., Guo S.H., Shang X.J., Yu L.S., Zhu J.W., Zhao A., Zhou Y.F., An G.H., Zhang Q., Ma B. (2018). Triptolide induces Sertoli cell apoptosis in mice via ROS/JNK-dependent activation of the mitochondrial pathway and inhibition of Nrf2-mediated antioxidant response. Acta Pharmacol. Sin..

[14] Matsuda Y., Watanabe T. (2003). Effects of oyster extract on the reproductive function of zinc-deficient mice: Bioavailability of zinc contained in oyster extract. Congenit. Anom..

[15] Zhang X., Qin X., Gao J., Lin H., Zhang C., Huang Y. (2019). Optimization of enzymatic hydrolysis from Crassostrea gigas and effects of its enzymatic hydrolysate on TM3 Leydig cells. J. Guangdong Ocean. Univ..

[16] Wang Q., Li W., He Y., Ren D., Kow F., Song L., Yu X. (2014). Novel antioxidative peptides from the protein hydrolysate of oysters (Crassostrea talienwhanensis). Food Chem..

[17] Li W., Xu C., Zhang C.H., Cao W.H., Qin X.M., Gao J.L., Zheng H.N. (2019). The purification and identification of immunoregulatory peptides from oyster (Crassostrea hongkongensis) enzymatic hydrolysate. Rsc. Adv..

[18] Liu Z.Y., Dong S.Y., Xu J., Zeng M.Y., Song H.X., Zhao Y.H. (2008). Production of cysteine-rich antimicrobial peptide by digestion of oyster (Crassostrea gigas) with alcalase and bromelin. Food Control.

[19] Umayaparvathi S., Arumugam M., Meenakshi S., Drager G., Kirschning A., Balasubramanian T. (2014). Purification and Characterization of Antioxidant Peptides from Oyster (Saccostrea cucullata) Hydrolysate and the Anticancer Activity of Hydrolysate on Human Colon Cancer Cell Lines. Int. J. Pept. Res. Ther..

[20] Miao J., Liao W., Kang M., Jia Y., Wang Q., Duan S., Xiao S., Cao Y., Ji H. (2018). Anti-fatigue and anti-oxidant activities of oyster (Ostrea rivularis) hydrolysate prepared by compound protease. Food Funct..

[21] Byun J.H., Choi Y.J., Choung S.Y. (2016). Protective effect of Oyster hydrolysate peptide in alcohol induced alcoholic fatty liver in SD-rats. Planta Med..

[22] Li S.J., Song Z.Y., Liu T.T., Liang J., Yuan J., Xu Z.C., Sun Z.H., Lai X.P., Xiong Q.P., Zhang D.Y. (2018). Polysaccharide from Ostrea rivularis attenuates reproductive oxidative stress damage via activating Keap1-Nrf2/ARE pathway. Carbohyd. Polym..

[23] Li Y., Qiu W., Zhang Z., Han X., Bu G., Meng F., Kong F., Cao X., Huang A., Feng Z. (2020). Oral oyster polypeptides protect ovary against d-galactose-induced premature ovarian failure in C57BL/6 mice. J. Sci. Food Agric..

[24] Bahadorani M., Tavalaee M., Abedpoor N., Ghaedi K., Nazem M.N., Nasr-Esfahani M.H. (2019). Effects of branched-chain amino acid supplementation and/or aerobic exercise on mouse sperm quality and testosterone production. Andrologia.

[25] Wu G. (2016). Dietary protein intake and human health. Food Funct..

[26] Leahy T., Gadella B.M. (2011). Sperm surface changes and physiological consequences induced by sperm handling and storage. Reproduction.

[27] Dong H.-J., Wu D., Xu S.-Y., Li Q., Fang Z.-F., Che L.-Q., Wu C.-M., Xu X.-Y., Lin Y. (2016). Effect of dietary supplementation with amino acids on boar sperm quality and fertility. Anim. Reprod. Sci..

[28] Sarmadi B.H., Ismail A. (2010). Antioxidative peptides from food proteins: A review. Peptides.

[29] Neto F.T., Bach P.V., Najari B.B., Li P.S., Goldstein M. (2016). Spermatogenesis in humans and its affecting factors. Semin. Cell Dev. Biol..

[30] Huynh P.N., Hikim A.P., Wang C., Stefonovic K., Lue Y.H., Leung A., Atienza V., Baravarian S., Reutrakul V., Swerdloff R.S. (2000). Long-term effects of triptolide on spermatogenesis, epididymal sperm function, and fertility in male rats. J. Androl..

[31] Bisht S., Faiq M., Tolahunase M., Dada R. (2017). Oxidative stress and male infertility. Nat. Rev. Urol..

[32] Zhang J., Bao X., Zhang M., Zhu Z., Zhou L., Chen Q., Zhang Q., Ma B. (2019). MitoQ ameliorates testis injury from oxidative attack by repairing mitochondria and promoting the Keap1-Nrf2 pathway. Toxicol. Appl. Pharmacol..

[33] El-Demerdash F.M., Yousef M.I., Kedwany F.S., Baghdadi H.H. (2004). Cadmium-induced changes in lipid peroxidation, blood hematology, biochemical parameters and semen quality of male rats: Protective role of vitamin E and beta-carotene. Food Chem. Toxicol..

[34] Morimoto H., Iwata K., Ogonuki N., Inoue K., Atsuo O., Kanatsu-Shinohara M., Morimoto T., Yabe-Nishimura C., Shinohara T. (2013). ROS are required for mouse spermatogonial stem cell self-renewal. Cell Stem. Cell.

[35] Zhou C., Hu J., Ma H., Yagoub A.E., Yu X., Owusu J., Ma H., Qin X. (2015). Antioxidant peptides from corn gluten meal: Orthogonal design evaluation. Food Chem..

[36] Zou T.B., He T.P., Li H.B., Tang H.W., Xia E.Q. (2016). The Structure-Activity Relationship of the Antioxidant Peptides from Natural Proteins. Molecules.

[37] Wajda A., Lapczuk J., Grabowska M., Slojewski M., Laszczynska M., Urasinska E., Drozdzik M. (2016). Nuclear factor E2-related factor-2 (Nrf2) expression and regulation in male reproductive tract. Pharmacol. Rep..

[38] Griswold M.D. (1998). The central role of Sertoli cells in spermatogenesis. Semin. Cell Dev. Biol..

[39] Geng X., Shao H., Zhang Z.H., Ng J.C., Peng C. (2015). Malathion-induced testicular toxicity is associated with spermatogenic apoptosis and alterations in testicular enzymes and hormone levels in male Wistar rats. Environ. Toxicol. Phar..

[40] Sadik N.A. (2008). Effects of diallyl sulfide and zinc on testicular steroidogenesis in cadmium-treated male rats. J. Biochem. Mol. Toxicol..

[41] Zhang X.Y., Qin X.M., Lin H.S., Cao W.H., Zheng H.N., Gao J.L., Zhang C.H. (2021). Protective effect of hydrolyzed ultrafiltration fractions from the Oyster (Crassostrea hongkongensis) on oxidative damage of TM4 Sertoli cells. South China Fish. Sci..

[42] Jiang X., Zhu C., Li X., Sun J., Tian L., Bai W. (2018). Cyanidin-3- O-glucoside at Low Doses Protected against 3-Chloro-1,2-propanediol Induced Testis Injury and Improved Spermatogenesis in Male Rats. J. Agric. Food Chem..

[43] Bonde J.P., Flachs E.M., Rimborg S., Glazer C.H., Giwercman A., Ramlau-Hansen C.H., Hougaard K.S., Hoyer B.B., Haervig K.K., Petersen S.B. (2016). The epidemiologic evidence linking prenatal and postnatal exposure to endocrine disrupting chemicals with male reproductive disorders: A systematic review and meta-analysis. Hum. Reprod Update.

[44] Pitteloud N., Dwyer A.A., DeCruz S., Lee H., Boepple P.A., Crowley W.F., Hayes F.J. (2008). Inhibition of luteinizing hormone secretion by testosterone in men requires aromatization for its pituitary but not its hypothalamic effects: Evidence from the tandem study of normal and gonadotropin-releasing hormone-deficient men. J. Clin. Endocr. Metab..

[45] Li M., Zhou M., Wei Y., Jia F., Yan Y., Zhang R., Cai M., Gu R. (2020). The beneficial effect of oyster peptides and oyster powder on cyclophosphamide-induced reproductive impairment in male rats: A comparative study. J. Food Biochem..

[46] Qian Z.J., Jung W.K., Byun H.G., Kim S.K. (2008). Protective effect of an antioxidative peptide purified from gastrointestinal digests of oyster, Crassostrea gigas against free radical induced DNA damage. Bioresour. Technol..

[47] Peng Z., Chen B., Zheng Q., Zhu G., Cao W., Qin X., Zhang C. (2020). Ameliorative Effects of Peptides from the Oyster (Crassostrea hongkongensis) Protein Hydrolysates against UVB-Induced Skin Photodamage in Mice. Mar. Drugs.

[48] Hamza R.Z., Al-Harbi M.S., El-Shenawy N.S. (2017). Ameliorative effect of vitamin E and selenium against oxidative stress induced by sodium azide in liver, kidney, testis and heart of male mice. Biomed. Pharm..

[49] Qiu C., Cheng Y. (2019). Effect of Astragalus membranaceus polysaccharide on the serum cytokine levels and spermatogenesis of mice. Int. J. Biol. Macromol..

[50] Oghbaei H., Hamidian G., Alipour M.R., Alipour S., Keyhanmanesh R. (2020). The effect of prolonged dietary sodium nitrate treatment on the hypothalamus-pituitary-gonadal axis and testicular structure and function in streptozotocin-induced diabetic male rats. Food Funct..

